# Molecular and Cellular Basis of Autoimmune Diseases

**DOI:** 10.3390/cells10020474

**Published:** 2021-02-23

**Authors:** Yasmina Juarranz

**Affiliations:** Departamento de Biología Celular, Facultad de Biología, Instituto de Investigación Sanitaria Hospital 12 de Octubre (imas12), Universidad Complutense de Madrid, 28040 Madrid, Spain; yashina@ucm.es; Tel.: +34-913945084

The defense organization of our organism is found in the immune system, which has two important components, the innate and the adaptive immunity, where different molecules, cells, and organs are involved and coordinated to protect us from external and internal damage. A major malfunction of the immune system occurs when it recognizes the auto-antigens in our own molecules, cells, or structures as a result of inadequate immune tolerance [1]. It recognizes them as harmful to us, inducing a cascade of molecular and cellular events involving the destruction of our own tissues, and thus we speak of autoimmune diseases. Their actual prevalence is not exactly known; however, some estimates indicate that more than 20% of the population suffers from one of these pathologies [2]. Therefore, many studies in bio sanitary research have as objective different molecular and cellular mediators in autoimmune diseases. Although there are common features in the different autoimmune diseases, it is important to note that there is a great deal of variability among them. Even within the same disease, there is high variability both in the development of the disease and in the response to different therapies [3]. Research in this field focuses on identifying the origin, events, and mediators of diseases and, consequently, finding molecular targets for the treatment of these diseases. Furthermore, given their diversity, the definition of biomarkers to predict the development of the disease or the response to treatment is also crucial.

This special issue in *Cells* contains 17 original and review articles linked to various novel aspects of autoimmune diseases. These articles address issues related to the involvement of innate or adaptive immunity, the influence of genetic variants, the microenvironment, and the importance of biomarkers or new therapies for these pathologies. Papers in this special issue can be grouped into two sections. The first discusses the influence of certain elements and mediators that are altered in these pathologies, and the second focuses on the search for biomarkers and appropriate therapies for autoimmune diseases.

In the first section and regarding the innate mediators implicated, an important original paper is published related to the implication of a macrophage subset-specific marker. Samaniego et al. describe the expression and regulation of the macrophage-specific Folate receptor β (FRβ) in tissue-resident macrophages, which is validated as a therapeutic target in several pathologies, including autoimmune diseases, such as rheumatoid arthritis [4]. The presence of this receptor in macrophages is associated with an anti-inflammatory profile of M-CSF-dependent IL-10-producing human macrophages (M-MØ) and strongly correlates with the presence of the PU.1 transcription factor. They address the macrophage expression of this receptor in rheumatoid arthritis (RA), where these cells preferentially exhibit a pro-inflammatory polarization. They found that although the expression of FOLR2 diminishes in macrophages from synovial membranes and synovial fluid in RA, FRβ is still detectable in the lining layer of the synovial membrane of RA patients. These results are compatible with the hypothesis that FRβ is a sensor for adjusting macrophages effector functions to extracellular folic acid levels and demonstrate the potential of this receptor as a therapeutic target.

Once innate immunity is triggered, adaptive immunity is activated, which is responsible for the extension and persistence of an autoimmune disease. In this sense, the modulation of the adaptive immune response can range from alterations in the development of a correct intrathymic selection of T lymphocytes to the development and imbalance of different Th cell subpopulations in the periphery. In this special issue, an in-depth perspective on several of these influences with one review and four original papers are provided. In the paper presented by Garcia-Ceca et al., they assess the indispensable interactions between thymic epithelial cells (TECs) and thymocytes (T) to perform a correct intrathymic T-cell education, but they also describe original results for this subject [5]. They describe the origin of thymic epithelium, the correlation between the cortical epithelium and the positive selection of T lymphocytes, the implication of TECs in both negative selection and Treg cell generation and, how Eph and their ligands, the ephrins, are implicated in cell attachment/detachment and govern, therefore, TEC–T interactions. In addition, they present original results that evaluated in vivo how many TECs would be necessary for supporting a normal T-cell differentiation, concluding that a significantly low number of TEC are still capable of supporting normal T lymphocyte maturation, whereas, with fewer numbers, T-cell maturation is not possible.

Another essential feature during the development of an adaptive immune response is the movement of leukocytes to areas of inflammation and the development of autoimmune pathologies. Oulghazi et al. describe the importance of α4-integrin (CD49d) in a mouse model for human type-1 diabetes (T1D), the spontaneously diabetic “non-obese diabetic” (NOD) mouse model [6]. They generate NOD mice with α4-deficient hematopoiesis (NOD.α4^−/−^) to study the role of α4 integrin in T1D. Their results indicate that these mice are protected from autoimmune diabetes and insulitis despite developing adaptive immunity, albeit attenuated, against islet cell autoantigens. Indeed, the authors demonstrate for the first time the critical dependence on α4 integrin for the development of autoimmune sialitis in NOD mice. From a translational point of view, α4 is shown as a potential target for primary or secondary prevention of T1D and could have immediate translational potential, since it is relatively safe, tolerable, and a highly effective α4-targeting reagent of pharmaceutical quality, i.e., the anti-functional anti-α4-antibody Natalizumab, are readily available. On the other hand, there is a reciprocal relationship between autoimmunity and the alterations of microbiota (Knip et al., 2016). In this study, Oulghazi et al. thus suggest that in the model presented here, abnormal microbiota are a consequence, not a cause of diabetes in NOD mice.

The Th17 subset has a key role in host defense against pathogens but also, Th17 cell-mediated inflammation, under certain conditions, is involved in the development of several autoimmune disorders, such as RA, Crohn’s disease, or systemic lupus erythematosus, promoting ongoing inflammation. Indeed, this subset is one of the most heterogeneous with plastic cells among the different Th subsets, and these facts are closely related to autoimmune diseases [7,8,9]. In this special issue, two manuscripts describe the association between the Th17 subset and autoimmunity. In the first, Salkowska et al. identify novel molecular markers of human Th17 cells using transcriptomic profiling and corroborating by other techniques [10]. They describe that the expression of the *APOD, C1QL1,* and *CTSL* genes in human CD4+ cells is restricted to Th17 cells and associated with high levels of acetylated histone H2BK12 at the promoter regions of these genes. These new markers will help us to identify better pathological conditions of clinical significance caused by Th17 cells. In the second article related to Th17, Sato et al. expose the implication of Fli-1 transcription factor in Th17 recruitment in a mouse model of Lupus Nephritis [11]. This transcription factor regulates the expression of numerous cytokines and chemokines and modulates the production of critical mediators associated with lupus nephritis in humans and in the MRL/lpr mouse model. Sato et al. demonstrate that the partial deletion of Fli-1 in this mouse model reduced IL-17, IL-6, IL-1β, and STAT3 expressions in renal tissue, and consequently, it could decrease the presence of Th17 cells during the development of renal inflammation.

The other component of adaptive immunity, humoral immunity, also has a main role in almost all autoimmune diseases. Its dysregulation may affect several structures and processes, such as antigen/antibody/Fc receptor complex formation, which is the case for autoimmune thyroid diseases (AITD). The pathogenesis of these diseases are poorly known, and the underlying autoimmune signature associated with them remains unclear. Martin et al. investigate in their original article the crosstalk between immune cell traits, secreted proteins, genetic variants, and the glycosylation patterns of serum IgG in a multi-omic and cross-sectional study in a big cohort of patients diagnosed with AITD [12]. They identify the glycan structures on antibodies, a subpopulation of immunoreactive NK cells, the secretion of Caspase-2 and IL-1α as immune features that are indicators detectable in the bloodstream of AITD status. They could be used in clinical situations in addition to the current tools, the thyroid-stimulating hormone (TSH) and thyroid peroxidase antibodies (TPOAb) levels, to understand the pathogenesis of these diseases better.

The microenvironment that surrounds immune cells is essential for determining their behavior, their response to resolve inflammation, and, therefore, to control the development of autoimmune pathology. Among the mediators present in the immune microenvironment are cytokines, chemokines, metabolic factors, neuropeptides, hormones, or pathogens. Adipokines are adipose tissue-derived factors that play a main role in metabolism but also modulate other central processes, such as inflammation. In autoimmune diseases, adipokines, such as adiponectin, leptin, visfatin, restin, and others, are involved in inflammatory pathways affecting different cell types. In addition, adipokines are able to interact and in part induce each other, forming a complex adipokine network. In this special issue, Neumann et al. review the most recent findings on the role of adipokines in the pathophysiology of inflammatory arthritis with a special focus on rheumatoid arthritis [13]. The authors describe the difficulty in assessing a specific role for each adipokine in a multifactorial disease, such as RA, since some of them have partial anti- or pro-inflammatory functions and could form complex adipokine networks.

Neuropeptides are present in the microenvironment of the immune cells; some of them are released by nerve endings but also secreted by the immune cells, such as the vasoactive intestinal peptide (VIP), which acts as an anti-inflammatory and immunomodulatory mediator, modulating the different Th subsets [14,15,16]. Indeed, several studies indicate a clinical approach for VIP and its receptors in several anti-inflammatory and autoimmune diseases [17]. In this special issue, Villanueva-Romero et al. analyze the senescent Th biomarkers in healthy donors and early arthritis patients and examine the bidirectional influence between the VIP axis and senescent Th cells. These pro-inflammatory cells are characteristic of immunosenescence but also several autoimmune/inflammatory diseases [18]. The paper describes that patients who meet classification criteria for RA have a higher percentage of CD4^+^CD28^−^ than those with undifferentiated arthritis. Thus, the senescence of Th cells induces a change in pattern expression of VIP receptors, and, in the other direction, VIP modulates some senescent Th biomarkers trying to counteract the senescence in these cells.

Hormones are also present in this microenvironment, and there is a clear mutual influence affecting common behavior, the endocrine system, and the immune system. Consequently, the vast majority of autoimmune diseases are affected by hormone levels, for example, sexual hormones. Systemic lupus erythematosus (SLE) typically affects young women at reproductive age, and this female predominance has been attributed to the immunostimulatory properties of hormones, such as prolactin (PRL) [19]. The results provided by Flores-Hernández et al. further underlines this fact. Using a mouse model of SLE, they support a mechanism in which PRL participates in this pathology through the rescue of self-reactive immature B cell clones from central tolerance clonal deletion through the activation of STAT3 and transcriptional regulation of a complex network of genes related to apoptosis resistance [20].

Associations among immune cells and other environmental factors, such as viruses, vitamins, or microbiota, have also been reported, and by extension, with autoimmune pathologies. The paper presented by Dominguez-Mozo et al. analyzes the humoral response to some virus infection, the levels of 25-hydroxyvitamin D (25(OH)D), and three main short-chain fatty acids (SCFA) related to bacterial metabolism from microbiota in multiple sclerosis patients [21]. Comparing these patients with healthy controls, they found significant differences in these environmental factors, supporting their possible involvement in the disease. Indeed, 25(OH)D and SCFA showed a negative correlation with Th17 cells and CD8^+^ cells in untreated MS patients.

In the second section of this Special Issue, we focus on the search for biomarkers and appropriate therapies for autoimmune diseases. A biomarker is a biological characteristic that can be used as an indicator of a biological state, such as illness, activity, or severity. Proteins, DNA, RNA, gender, or age have been proposed as valuable biomarkers [22]. They help us resolve the uncertainties that a physician encounters when visiting autoimmune diseases in patients regarding diagnosis, prognosis, activity, or response to treatment. In this special issue, three papers provide in-depth studies on the search for genetic, epigenetic, or protein biomarkers in several diseases, such as juvenile idiopathic arthritis, RA, psoriasis, or autoimmune thyroid disease. Two of the manuscripts are remarkable reviews. In the first, Hou et al. describe the utility of performing a multi-omics study to understand the heterogeneity and complexity of juvenile idiopathic arthritis (JIA) [23]. Thanks to genetic studies of monogenic or polygenic forms, transcriptome studies in different immune cells, and epigenomic studies of JIA, we could have a new molecular knowledge of different JIA subtypes, and as a consequence, an improvement in the classification of this pathology and the treatments that should be used in each subtype to obtain a better response. In the second, Wysocki et al. perform an in-depth review on the *HLA-DRB1* gene, linked to the development of several autoimmune diseases, but strongly associated with RA and the presence of anti-citrullinated protein antibody (ACAP) that indicates its importance in RA morbidity [24]. In addition, they explain that different haplotypes of *HLA-DRB1* are associated with the occurrence of non-articular disease manifestations or different responses to any RA treatment. So the *HLA-DBR1* gene would be crucial for the development of genotype-matched diagnostic and treatment protocols in RA patients.

The other is an original article addressing this issue in one autoimmune pathology, psoriasis. Micro RNAs (miRNAs) are highly conserved small non-coding RNAs that act as regulators of gene expression [25]. In the last ten years, many studies have been performed to understand the role and implication of these molecules, and it has been reported that several situations or body conditions modify miRNAs expression, such as sex, age, lifestyle factors, or inflammation. In this sense, different miRNAs expression profiles are found in autoimmune diseases and propose potential miRNA signatures to predict outcome or response to therapy, thus underlining the role of these molecules as clinical biomarkers. Chicharro et al. propose in their article that one miRNA, miR-135b, could be used as a prognostic biomarker in psoriasis since its basal levels in lesional skin could help to identify those patients that will improve after treatment with biologics, regardless of the therapeutic target used [26].

Current therapies for autoimmune diseases can be classified into two main clusters, therapies that only control symptoms: the non-steroidal anti-inflammatory drugs (NSAIDs) or corticoids, and therapies that change the natural outcome of the disease and, as a consequence, slow down the damage and even reverse the disease. These last therapies are the disease-modifying drugs (DMDs), which can be subdivided into two groups non-biological or synthetics DMDs (conventional synthetic DMDs or csDMDs and target synthetic DMDs or tsDMDs), and biological DMDs. In this special issue, four articles are presented that focus on this topic. Two address the development of new DMDs. The review of Galindo-Izquierdo et al. is about the usefulness of the complement system (CS) as a therapeutic target in systemic autoimmune diseases through the use of CS inhibitors as DMDs drugs [27]. The CS belongs to innate immunity, and its main function is to recognize and protect against foreign or damaged molecular components. In addition, other homeostatic functions of CS are the elimination of apoptotic debris, neurological development, and the control of adaptive immune responses [28]. However, the CS has a main pathogenic role in several systemic autoimmune diseases, such as SLE, antiphospholipid syndrome, Sjögren Syndrome, RA, or ANCA-associated vasculitis [27]. Although the development of CS inhibitors is still limited and complex, its role in systemic autoimmune diseases provides the pathogenic basis for the development of therapies designed to block certain facets of complement and could be included. Other interesting DMDs molecules are the tsDMDs which are a large family of small molecules targeting several types of kinases that are crucial in the downstream signaling of pro-inflammatory molecules. The review exposed by Massalska et al. highlighted the use of small molecule inhibitors targeting intracellular JAKs/MAPKs/NFκB/SYK-BTK signaling pathways for RA treatment, the changes associated in the patients, but also their risk and adverse effects [29]. Furthermore, due to their ability to block pro-inflammatory signaling pathways, the use of these tsDMDs is extended to fight other pro-inflammatory pathologies, such as COVID-19.

With respect to new NSAID molecules and, although the studies conducted by Liu et al. in the mice model of psoriasis are preliminary, (R)-Salbutamol could be considered in the NSAID group for the treatment of psoriasis after further study and extension to human treatment [30]. This molecule is a β_2_-adrenergic agonist widely used in asthma and, in addition, is able to reduce the symptoms of imiquimod-induced psoriasis in mice through the regulation of Th17/Treg cell response and glycerophospholipid metabolism.

To conclude the section on treatments for autoimmune diseases, the review presented by Lopez-Santalla et al. address a new type of treatment for autoimmune disease, cell therapy [31]. In recent years, mesenchymal stem/stromal cell (MSCs)-based therapies have largely been proposed as a novel and promising stem cell therapeutic approach in the treatment of some autoimmune pathologies, such as RA. These stem cells are multipotent progenitor cells that have immunomodulatory properties and are easy to obtain and expand. The review discusses MSC-based therapy approaches with a focus on published clinical data, as well as on clinical trials for treatment of RA that are currently underway.

Although the scientific field of autoimmune diseases is very open and there is still much to be done, this Special Issue provides a comprehensive update on the current molecular and cellular basis of autoimmune diseases both to understand the basic mechanism involved and also for a potential translation to the clinical practice.
